# The McMaster Optimal Aging Portal: Usability Evaluation of a Unique Evidence-Based Health Information Website

**DOI:** 10.2196/humanfactors.4800

**Published:** 2016-05-11

**Authors:** Angela M Barbara, Maureen Dobbins, R. Brian Haynes, Alfonso Iorio, John N Lavis, Parminder Raina, Anthony J Levinson

**Affiliations:** ^1^ Health Information Research Unit Department of Clinical Epidemiology and Biostatistics McMaster University Hamilton, ON Canada; ^2^ School of Nursing McMaster University Hamilton, ON Canada; ^3^ McMaster Health Forum Centre for Health Economics and Policy Analysis, Department of Clinical Epidemiology and Biostatistics, and Department of Political Science McMaster University Hamilton, ON Canada; ^4^ McMaster Institute of GeroScience Department of Clinical Epidemiology and Biostatistics McMaster University Hamilton, ON Canada; ^5^ Division of e-Learning Innovation Faculty of Health Sciences McMaster University Hamilton, ON Canada

**Keywords:** online health information, health informatics, elderly, consumer health information, qualitative research, usability testing, Internet, evidence-based medicine, knowledge translation, aging, website

## Abstract

**Background:**

Increasingly, older adults and their informal caregivers are using the Internet to search for health-related information. There is a proliferation of health information online, but the quality of this information varies, often based on exaggerated or dramatic findings, and not easily comprehended by consumers. The McMaster Optimal Aging Portal (Portal) was developed to provide Internet users with high-quality evidence about aging and address some of these current limitations of health information posted online. The Portal includes content for health professionals coming from three best-in-class resources (MacPLUS, Health Evidence, and Health Systems Evidence) and four types of content specifically prepared for the general public (Evidence Summaries, Web Resource Ratings, Blog Posts, and Twitter messages).

**Objective:**

Our objectives were to share the findings of the usability evaluation of the Portal with particular focus on the content features for the general public and to inform designers of health information websites and online resources for older adults about key usability themes.

**Methods:**

Data analysis included task performance during usability testing and qualitative content analyses of both the usability sessions and interviews to identify core themes.

**Results:**

A total of 37 participants took part in 33 usability testing sessions and 21 focused interviews. Qualitative analysis revealed common themes regarding the Portal’s strengths and challenges to usability. The strengths of the website were related to credibility, applicability, browsing function, design, and accessibility. The usability challenges included reluctance to register, process of registering, searching, terminology, and technical features.

**Conclusions:**

The study reinforced the importance of including end users during the development of this unique, dynamic, evidence-based health information website. The feedback was applied to iteratively improve website usability. Our findings can be applied by designers of health-related websites.

## Introduction

### Background

Increasingly, older adults and their informal caregivers are using the Internet to search for medical or health-related information [1-5]. Over two-thirds of people aged 65 years or older use the Internet daily [6,7]. Access to health information allows consumers to participate in their health decisions and increases patient satisfaction, health knowledge, and understanding of the benefits and risks of treatment [8-10]. However, these beneficial effects are predicated on the availability of practical, timely, and high-quality information.

There is a proliferation of scientific research posted on the Internet daily that often promotes exaggerated findings and conflicts with existing research. Online newspapers and media cover many health-related stories but the emphasis is often on dramatic findings from a single, new study [11] rather than a body of literature such as systematic reviews or meta-analyses [12,13]. A variety of online health resources are available, but the quality of the information varies across websites and is often poor [14,15]. Medical advice offered by for-profit companies and celebrities may not be scientifically sound and could be harmful [16-19]. Also, health information websites may not be senior friendly [20,21]. Lastly, most health websites focus on specific diseases or health conditions without providing a broader view of aging by including content on disease prevention or health promotion.

Seniors experience numerous challenges when they search for health information online. Low levels of health literacy (ability of individuals to gain access to, understand, and use information in ways that promote and maintain good health) [22] can interfere with consumer comprehension and use of scientific evidence [23-25]. More than half of online health information seekers do not check the source of the information that they find online [26], and many seniors do not know how to critically assess the reliability of a website, appraise the quality of the health information provided, or differentiate between scientific evidence and paid advertising [27,28]. The McMaster Optimal Aging Portal (Portal) was developed to provide Internet users with high-quality evidence from health research, addressing some of these current limitations [29].

Clinicians may make use of evidence-based health information for citizens as part of a self-management support strategy for patients [30]. Including high-quality, actionable, educational materials may promote patient self-management, although a systematic review of printed educational materials showed only small effects [31]. Public health professionals may use the Portal’s content for citizens as evidence-informed resources for public education on a variety of topics; this could be considered as part of a multifaceted knowledge translation strategy [32]. Nonprofit organizations and patient advocacy associations may use the Portal as an efficient broker of best evidence to support grant applications or evaluations. Policymakers may also engage with the Portal as a one-stop shop to guide evidence-informed decision making [33]. There may be an important role for government to play in facilitating access to high-quality health information similar to trends in large research-granting agencies that encourage open access to research publications [34].

Our vision for the Portal was to create a comprehensive, continuously updated, evidence-based health information website on optimal aging. We defined optimal aging as the maintenance of good health, physical activity, and engagement in life and the management of health conditions. The Portal targets citizens (ie, the general public) as well as clinical, public health, and policy professionals. The content is geared toward an international English-speaking audience and not restricted to any health care system.

Researchers have emphasized the importance and added value of input by intended users of consumer health websites in addition to expert review [35-39]. While the quality of online health information websites has been assessed by various methodologies including heuristic evaluation using published criteria, guidelines, or assessment measures, we wished to see if citizens were concerned with similar facets such as website content, design, disclosure of authors and developers, currency of information, and ease of use [20,40-42].

### Objectives

Our objectives were to evaluate and enhance the features of the website that were specifically geared toward citizens. We applied usability techniques to incorporate the experience and formative feedback of members of the target audience while engaging with the Portal. In this paper, we share the findings of the usability evaluation of the Portal focused on the content features for the general public to better inform designers of health information websites and online resources for older people and caregivers regarding key usability themes.

## Methods

### Project Background

McMaster University’s Labarge Optimal Aging Initiative has quickly established itself as a source of innovative research and trusted information for the benefit of the aging population. The Initiative continues to seed research projects intended to maximize the resilience of the older adults and has created the McMaster Optimal Aging Portal, a website that provides access to quality reviewed and understandable information for a variety of stakeholders.

### McMaster Optimal Aging Portal: Website Components

#### Home Page

**Figure 1:**
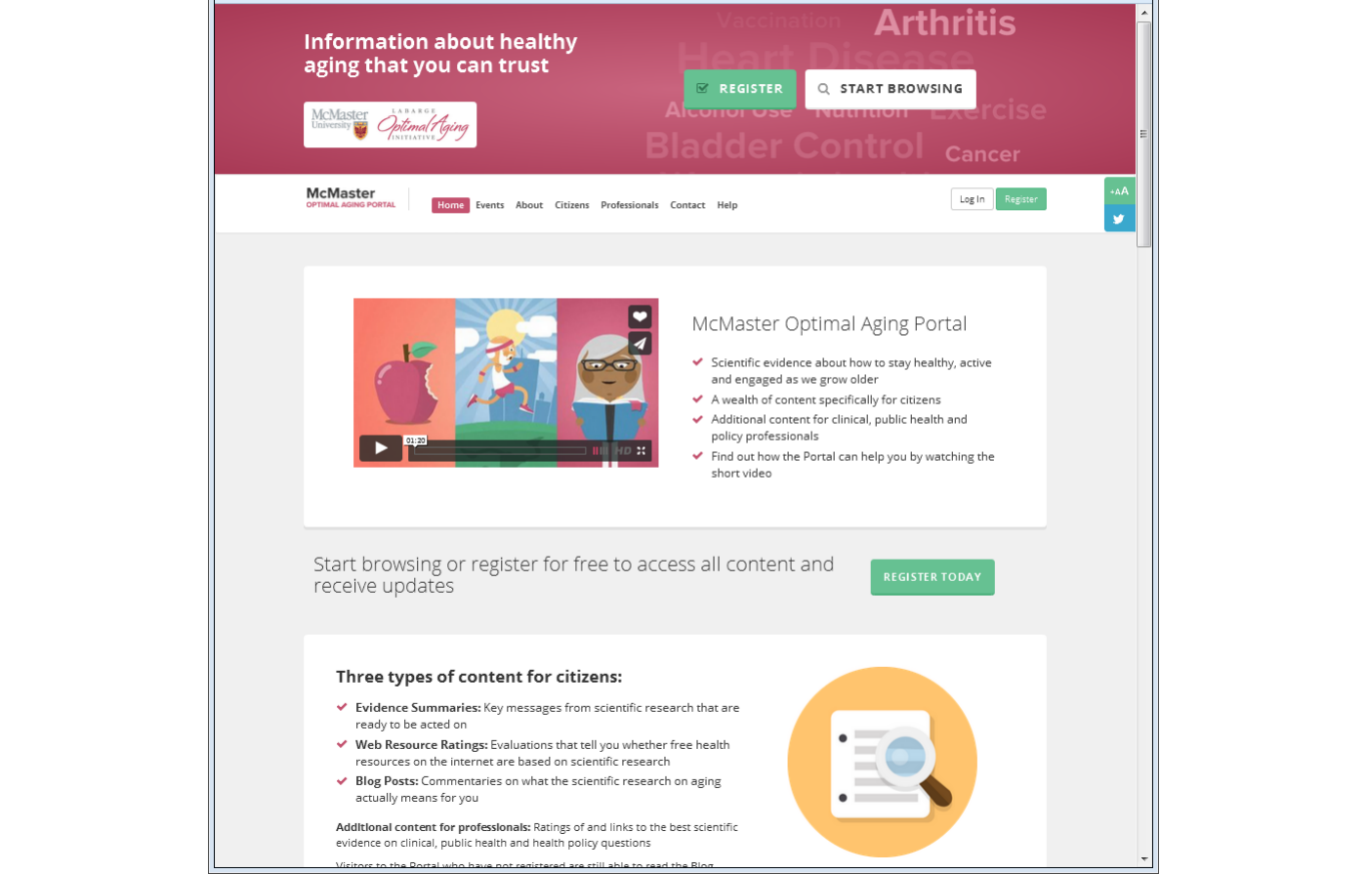
McMaster Optimal Aging Portal home page.

The home page provides an introduction to the Portal and includes a 90-second video (Figure 1). The main menu is organized by Home, Events, About, Citizens, Professionals, Contact, and Help. There are short descriptions of the types of content for citizens and links to the most recent content. At the bottom of the page is the Twitter feed providing links to research evidence and news articles relating to aging. The homepage was designed for users to have access to all of the components of the Portal. There are prompts to register, log in, and start browsing. A full description of the Portal design and development is available in Multimedia Appendix 1.

#### Evidence-Based Content for Citizens

##### Evidence Summaries

Evidence Summaries are short 1- or 2-page documents that describe in consumer friendly language the findings of a systematic review found in one of the three professional databases: McMasterPLUS for clinical content, HealthEvidence for public health, and Health Systems Evidence for health policy. When available, the Portal provides links to plain language summaries prepared by the Cochrane Collaboration, a global network that works to make sure health research is useful and accessible [43], with titles that emphasize the key message from the research evidence.

##### Web Resource Ratings

Web Resource Ratings are systematic evaluations to assess the quality of existing third-party Web-based health information. The Portal team searches for health-related websites that are relevant to aging, not directly funded by a company trying to sell products or services, intended for or including content intended for citizens, and freely accessible. The team then looks for specific resources on the website such as videos, fact sheets, articles, health calculators, and online quizzes. To be added to the Portal, Web resources must meet the same inclusion criteria as the websites and also be current (updated within the last 5 years). These Web resources are evaluated for quality of the evidence, description of the resource’s development, and usability using a star system from 0 to 5, where 0 = lowest possible, 1 = information is not based on evidence and we do not recommend, 3 = information is based on possibly only one or two studies and we recommend reading more about this topic, and 5 = information is reliable and we recommend discussing it with your health care professionals.

##### Blog Posts

Blog Posts provide easy-to-understand information in a narrative format based on the best available and most recent scientific evidence on topics that matter to older people. Blog Posts are short articles that integrate information from a variety of sources on health topics. They are written by a professional writer or expert on the topic, assessed for accuracy by an expert in interpreting and communicating the scientific literature, and edited by a professional editor. Each Blog Post includes bottom-line recommendations based on the best available scientific evidence.

##### Mac_Aging News

The Twitter feed offers one-sentence take-home messages from evidence briefs and systematic reviews related to optimal aging appearing in mainstream media on any given day. The tweets are about news followed immediately by the related evidence from the Portal (see Multimedia Appendix 1). The Twitter feed is also used for disseminating updates related to knowledge translation activities and to stimulate discussions during live Web streaming of public talks on topics related to optimal aging.

#### Design and Development

In developing the infrastructure, we were mindful of functional changes related to aging that can affect computer use by older adults [44]. For example, to address vision impairment we used adjustable font size and dark type against a light background. We also adhered to Web development guidelines for creating websites that work well with older adults [45-48].

#### Registration

The Portal can be browsed without a user account but registration is required to perform a search and access all of the content. During registration, users must select a role or personal category (citizen, clinician, public health professional, or policymaker), which allows us to personalize the content. There is no restriction on access to content once someone has registered; citizens are able to access all professional content and vice versa. Registrants can also opt to receive email alerts that contain links to newly prepared Evidence Summaries, Web Resource Ratings, and newly identified research specific to selected topics for professionals.

#### Navigation and Content Retrieval

The Portal’s overall organization, page design, font, icons, and links have been designed and constructed to afford the user ease of navigation to its many features. The search engine powers the retrieval of Portal content with features that both categorize and prioritize its search results. The Portal also offers an option to browse the 66 unique topics organized into 3 categories: health conditions, healthy aging practice, and health care delivery. Browsing is characterized by its exploratory nature and absence of planning, goals, or objectives [49], whereas searching is goal directed with the user looking for specific information to solve a problem or fulfill specific information needs [45,50].

### Website Usability Evaluation

#### Study Design

We used mixed methods of data collection encompassing usability testing and semistructured interviews to generate qualitative and semiquantitative data on user experience with the Portal. The study was conducted from July to September 2014, and the protocol was approved by the Hamilton Integrated Research Ethics Board. All participants provided informed consent.

#### Recruitment

Participants were required to have access to a computer or mobile device with an Internet connection and belong to at least one of our two target user groups: (1) aged 50 years or older and not employed as a clinician, public health worker, or policymaker or (2) informal caregiver (person who provides unpaid care to a parent, family member, friend, or loved one) of any age. We planned to recruit 8 to 12 individuals in each user group and for each type of testing (usability testing and individual interviews), as suggested by Hwang and Salvendy [51], and ceased recruitment once data saturation was reached [52]. We recruited participants through the formal and informal networks of the Portal working group. The study was advertised in the subscription HealthEvidence newsletter. Advertisements were also posted at local senior organizations and community bulletin boards. Fliers were distributed to members of senior groups at McMaster University and at community events focused on healthy aging. Usability testers were invited to participate in the focused interviews and vice versa. We aimed to recruit a sample that was diverse in age, gender, and health status.

#### Usability Testing

##### Overview

Usability testing sessions took place either in person, by telephone, or using a videoconferencing application (Skype) based on participant preference, geographical proximity, and logistical or physical mobility limitations. In-person interviews took place in the usability lab on the McMaster University campus with a desktop computer. For telephone and Skype sessions, participants were asked to choose a quiet location free of distractions, preferably at home or work and not in a public area (such as a library or coffee shop).

Researchers have argued that the use of videoconferencing technologies is a viable complement or replacement for in-person qualitative interviews with the benefits of time efficiency, scheduling flexibility, and reduced cost [53-55]. For Skype sessions, we used videocalling and instructed participants to click on the Share Screen feature. This allowed the facilitator to see the participants and their computer screens synchronously. Those testing over the telephone were sent the Portal website address by email before the session. Participants were instructed and prompted to say what they were looking at and what they were doing or trying to do (eg, clicking on a link, scrolling down the Web page, looking at content, reading, entering text) so that the facilitator was able to mimic their actions and follow along on her computer screen.

Participants were emailed the consent form to review in advance of the session. Those who participated in person brought the signed and witnessed form with them to the session or signed a form in front of the facilitator. Remote (telephone and Skype) testers provided audiorecorded verbal consent or mailed a signed consent form to the facilitator.

Participants chose which Web browser to use based on their experience and comfort level. The facilitator followed a usability guide that we specifically developed for citizens based on the work by Steve Krug [56,57]. We used two usability techniques during the sessions: direct observation and task completion.

##### Direct Observation

Participants were invited to look first at the home page and then explore the Portal while employing the think-aloud method in which they verbalized what they were doing, thinking, and feeling [58,59]. The facilitator prompted the participants to explain their actions and expectations. At the start of the session and while the participants were using the Portal, they were encouraged to verbally express their initial impressions of the Web pages, perceptions about ease of use, what they liked and disliked about the website, and what could be improved.

##### Task Completion

To identify specific features and issues with the interface, participants were asked to perform tasks focused on registration, content, and navigation. The facilitator provided help only when the participant reached a roadblock. Participant performance was assessed by task completion and frequency of assistance. Using the benchmark developed by Rubin and Chisnell [60], if more than 70% of participants were not able to complete a task without help, it was classified as a usability problem requiring attention to remedy.

For the live sessions, the facilitator observed the participant’s actions and physical cues throughout testing to assess task completion. During the Skype sessions, the facilitator was able to observe by video the participant’s actions on the screen. The facilitator used a slower pace throughout the session, repeated questions, confirmed participant responses, paid particular attention to facial expressions, and allowed for “pauses” in technology [61]. For telephone interviews and in the absence of nonverbal cues, the facilitator was dependent on tone of voice and what was communicated aloud. Prompts were used if the participant was silent: Please keep talking. What are you looking at? What are you thinking? What are you doing now? Is that what you expected [56]?

In keeping with the iterative process, the usability script was revised based on user feedback or when unexpected usability issues arose. Thus, the script evolved during the course of testing.

#### Focused Interviews

We conducted individual interviews to evaluate the usability of 3 of the 4 features of the Portal tailored for citizens (Evidence Summaries, Web Resource Ratings, and Blog Posts). Interviews were done either in person, by telephone, or using Skype, based on the preference of the participant. Individuals were invited to evaluate one feature of the Portal during the interview.

Participants were asked to review a minimum of either 3 Evidence Summaries, 2 Web Resource Ratings, or 2 Blog Posts. We used the think-aloud method as users read through the content followed by a semistructured interview guide to elicit further feedback about the user experience. The interview script was guided by Morvilles’ user experience honeycomb, which states that a user will have a meaningful and valuable experience if the information is findable, accessible, desirable, usable, valuable, useful, and credible [62].

At the end of each usability session and interview, the facilitator asked a series of questions to obtain demographic data (age, employment, health status, education, and personal income).

#### Data Analysis

All the usability sessions and interviews were audiotaped and transcribed verbatim. The usability testing and interview data were analyzed together. This triangulation approach [63] allowed us to obtain a broad review of the website’s usability. Data analysis was inductive rather than theory-based because we sought to understand participant experiences with the Portal. We used qualitative content analysis [64]. Two authors (AMB, AJL) independently read the transcripts and attributed codes to phrases or paragraphs (open coding) and developed the coding schema. The data were organized into core themes, paying particular attention to facilitators and barriers to favorable user experiences. We applied axial coding to develop connections among the coding categories. The qualitative software program NVivo 9 (QSR International) was used for coding the data.

## Results

### Participant Characteristics

Of the 63 individuals responding to advertising about the study, 37 (59%) participated in at least one testing session (Table 1). Reasons for nonparticipation included professional status (eg, public health worker, clinician; 17/26, 65%), scheduling conflicts (7/26, 27%), or nonuse of computer (2/26, 8%).

Our sample included 22 women and 15 men ranging in age from 23 to 84 years (mean 69 years); 11 were informal caregivers. Computer experience varied; on average, users spent an estimated 16 hours per week online (range 2-55 hours), including checking emails and browsing the Internet. More than half of users (57%) spent at least 14 hours per week online. Some individuals (especially younger users and those who used computers for their current or previous work) were computer savvy; others required assistance with the Web browser or computer-specific actions such as returning to the previous page, recognizing and opening hyperlinks or new browser windows/tabs, and accessing email on an unfamiliar computer. All users had Internet access at home; 68% had a desktop computer, 29% had a tablet, and 27% used a laptop (6 had more than one type of computer at home).

**Table 1:** Participant characteristics.

		n (%)
Age category		
	49 years or younger	2 (5)
	50-59 years	4 (11)
	60-69 years	12 (32)
	70-79 years	14 (38)
	80 years and older	5 (14)
Gender		
	Female	22 (60)
	Male	15 (40)
Ethnic group		
	White	34 (92)
	Black/African Canadian	2 (5)
	Asian	1 (3)
Employment status		
	Retired	28 (76)
	Semiretired	3 (8)
	Full-time work	4 (11)
	Part-time work	1 (3)
	Unemployed	1 (3)
Caregiver		11 (30)
Health status		
	No medical conditions	14 (38)
	One or more medical conditions	23 (62)
Education		
	High school grad or less	2 (5)
	Some college/university	2 (5)
	College/university graduate	20 (54)
	Some postgraduate or more	13 (35)
Personal income		
	Less than $39,000	11 (30)
	$40,000 to $59,999	13 (35)
	$60,000 to $79,999	4 (11)
	$80,000 or more	4 (11)
	Prefer not to answer	5 (14)

**Table 2:** Testing characteristics.

		n (%)
Participation		
	Usability testing only	17 (46)
	Usability testing and focused interview	16 (43)
	Focused interview(s) only	3 (8) ^a^
Setting		
	Laboratory, in-person	15 (41)
	Telephone	13 (35)
	Skype	9 (24)
Computer		
	Desktop	29 (78)
	Laptop	6 (16)
	Tablet	2 (5)
Browser		
	Internet Explorer	13 (35)
	Firefox	11 (30)
	Google Chrome	10 (29)
	Not sure	3 (8) ^b^

^a^Two participants each participated in 2 focused interviews.

^b^Remote testers.

### Study Setting

We conducted 33 usability testing sessions and 21 individual interviews (Table 2). Fifteen people participated in person and 13 by telephone. Of the 9 users who participated using Skype, 3 were using Skype for the first time and one user was unable to share his screen (due to technical difficulties). All testing sessions and interviews were conducted by one of the authors (AMB). Usability testing averaged 54 minutes (range 45-75 minutes). Interviews averaged 44 minutes (range 30-55 minutes). Most participants (34/37, 92%) had not visited the website before testing and were able to share their initial reactions.

### Usability Evaluation Findings

#### Task Performance

Performance on the 9 tasks is presented in Table 3. In summary, 4 tasks were completed easily by participants: finding where to register, completing the registration form, browsing content, and locating a Web Resource Rating. The remaining 5 tasks revealed some difficulties with usability: playing the introductory video, validating registration, searching, finding an Evidence Summary, and locating a Blog Post.

There were 9 participants who did not realize that the visual display on the home page was a video despite the text alongside it saying “Find out how the Portal can help you by watching the short video.” The Play button was hidden unless users hovered over the video, leading most people to assume it was a static picture. One participant opted out of watching the video.

I very rarely watch a short video. But I know a lot of people who will look at it.

User without medical conditions, age 59

Users immediately noticed the Register button on the home page. One user was unsure if one or two clicks of the mouse were required and another went to the log-in page instead. Users found the first part of the registration process—filling out the short form—easy and straightforward. However, the second part—completing the registration by clicking a verification link in an email message—was either tedious or problematic. Once the registration form was completed and the Register button was pressed, participants assumed they were registered and logged in. Others were not sure how to proceed. Some people did not read the text on the screen prompting them to check their email.

In general, users preferred to navigate the website by browsing rather than searching. Browsing allowed many users to explore what was in the Portal. Searching, however, was challenging for a third of users. Many participants were uncertain what to search for, either because they did not have a health topic in mind or were not sure what search terms to use; some wanted to know more about the website content first. In response to “How would you find something of interest to you on the Portal?” a few people said they would “Google it.” Therefore, query formation was based on their experience using Google and search strings tended to be very narrow, such as “male urinary incontinence,” “caring for disabled spouse,” or “lobular breast cancer.” At the time of testing, there was much less citizen content than professional content, so users who provided broader search terms such as “exercise” or “cancer” had more search results.

While many users were able to find an Evidence Summary, Web Resource Rating, or Blog Post on their own, some needed assistance and asked for help (Table 3). This was due mainly to their inexperience with the layout of the website or hesitation to click without confirmation that it was okay by the facilitator.

Due to the initial limitations of the quantity of citizen content, some users were able to find content but were categorized as unsuccessful at tasks related to information seeking because they were unhappy or frustrated by the results (ie, they did not find information they were interested in, expected, or wanted to find).

**Table 3:** Task performance findings.

Task	n	Completed with ease n (%)	Completed with help n (%)	Did not complete n (%)
Play video on the home page	20	10 (50)	9 (45)	1 (5)
Find where to register on the Portal	28	26 (93)	2 (7)	0
Complete the registration form	18	14 (78)	2 (11)	2 (11)
Validate the registration	17	7 (41)	7 (41)	3 (18)
Find content of interest by browsing	26	22 (85)	2 (8)	2 (8)
Find content of interest by searching	22	10 (45)	7 (32)	5 (23)
Find an Evidence Summary of interest	28	18 (64)	9 (32)	1 (4)
Find a Web Resource Rating of interest	28	22 (79)	5 (18)	1 (4)
Find a Blog Post of interest	25	12 (48)	11 (44)	2 (8)

#### Qualitative Analysis

##### Themes Related to the Benefits of the McMaster Optimal Aging Portal

The positive features of the website that emerged from the data related to its credibility, applicability, browsing function, design, and accessibility (Textbox 1).

The McMaster University logo gave the website immediate credibility. Users felt they could rely on the evidence because the Evidence Summaries provide reviews of new research based on best available scientific evidence, a service that is typically available only to professionals. The value of the Web Resource Ratings was that a trustworthy source had reviewed websites and their resources so that users could count on those assessments and use (or not use) the material with confidence. The Blog Posts were written by experts whose credentials were listed. The lack of advertising and product promotion was also appreciated by users.

One of the identified benefits of the website was its ability to provide current information about issues that mattered to participants, either to themselves personally or to older adults they were caring for. Users were also excited about finding information they could discuss with their health care provider. The content format of the information (eg, Evidence Summary, Blog Post) was not as important as the subject matter and its applicability to the participants’ own lives.

After reviewing the home page, participants were asked how they wanted to use the Portal. Most people wanted to start browsing immediately, even before they had seen everything on the home page. The browse function facilitated an exploratory approach. A few users said they would click on all the topics that interested them. The topic lists prompted some participants to tell stories about the personal or community impact of health conditions, healthy practices, or health care delivery. A few users said they would use the Browse Topics page as a landing page for navigating through the Portal.

Participants made positive comments about the aesthetics of the Web pages. The 90-second video on the home page provided a better introduction to the website than did the text. The images and titles of the Blog Posts were a big lure to users, especially if the topic (eg, yoga, medication safety) was of interest.

Participants liked that the home page was “basic” and “without too much stuff” to read through. In terms of the amount of information provided, users were happy with the concise nature of the Evidence Summaries and would have been unwilling to read beyond that length. Satisfaction with the information was also determined by its appearance and layout. That is, participants were content not to read long blocks of text; many admitted to being scanners or skimmers who did not read every single word but looked for pieces of information that were perceived as personally relevant. Overall, the most valuable pieces of information were the conclusions of the Evidence Summaries and “The Bottom Line” of the Blog Posts. One user’s comment represented the sentiments of the sample:

For me as a human being, all I care about is: Will this affect me? Will my life get easier? Is it going to hurt me?”

Caregiver, age 59

**Themes related to the benefits of the McMaster Optimal Aging Portal.**

Credibility: perceptions about trustworthiness of content, source of information

I do trust this [blog post] , because it was researched and it wasn’t just something I pulled up on the Internet like you know, when I do a search for something, you don’t know the credibility, but this is a research project from a university so yes, I do trust that. [User without medical conditions, age 81]

So after watching the video, it gives me more confidence in the website. Because now I am thinking it is more of a . . . professional site, like PubMed or a peer-reviewed journal or something like that. It would be a safe place to go to get some proper information for my grandparents, and it gave me a lot more confidence in the site and its legitimacy. [Caregiver, age 23]

Applicability: perceived use of information, relevance to self or others

I would look at stroke [topic]. I have never had a stroke but I have a friend of mine that recently had a stroke. So that would be interesting because that just happened last month or so. [User with medical conditions, age 79]

My immediate feeling is I would go to a website like this because either I’ve got a problem or a loved one has a problem. I would be under a certain amount of stress, if not a lot of stress. [User without medical conditions, age 75]

Browse function: ability to browse content organized by topic

Good variety of topics; comprehensive. Great list. I assume this is a map to all the topics that are covered. [User without medical conditions, age 62]

It is asking me if I want to start browsing and that is what I want to do. [User with medical conditions, age 61]

Design: visual appeal, first impression, sample content on homepage, images, video introduction on homepage

It seems straightforward, interesting visually. [User with medical conditions, age 70]

These are good articles; I would read each and every one of them. [Caregiver with medical conditions, age 59]

Oh, it is good. The video explains the purpose and how to use it very well. [User with medical conditions, age 82]

Accessibility: level of comfort with language, amount of information, readability

They [Evidence Summaries] do not get into a ton of details, but I think that that is what some people are looking for; they are just looking for a kind of a summary and recap. And it is nice to have a chart at the end to summarize everything. [Caregiver, age 23]

I found the information easy to understand and very straightforward. I mean, it is broken up into clear sections, and it is broken up into different underlines and bold words, so it is pretty easy to read. And it has got colors around to kind of break it up too. [Caregiver, age 23]

So I like the layout. How it is easily readable. [User without medical conditions, age 59]

##### Themes Related to Usability Challenges

A number of usability challenges emerged from the data: reluctance to register, process of registering, searching, terminology, and technical features (Textbox 2).

While about one-third of users felt that it was not unusual to register for an information website and would do so willingly, other participants voiced their surprise, disinclination, or apprehensions about registering. Before starting the registration process, users were uncertain why registration was needed and quite concerned about the provision and potential use of their personal information. Once on the registration form page, participants were content to provide the minimum amount of data requested (email address, country, and role). We observed that very few people read the information provided with respect to “Why register?” while on this page. Once a user decided to register, this material was no longer applicable.

Many older users were unfamiliar with the usual steps required for setting up a user account. As seen by the task analysis, the validation step was particularly challenging. Without guidance from the facilitator, many users would not have fully completed registration by checking their email for the validation link.

The qualitative analysis also confirmed that searching the website was difficult for many users. The challenge lay in query formation and search strategy. For query formation, users needed to know what was in the repository being searched and needed to match that information to their own interests. Many relied on their experience with Google when entering search terms, expecting such features as automatic word completion and spell check.

A number of users commented that they were comfortable with the language level; however they cautioned that it would be too high and not understood by the general public, especially the “intellectual jargon.” Most users were able to correctly describe an Evidence Summary but were uncertain or unfamiliar with the names Blog Post and Web Resource Ratings. A substantial number of users said they would not read blogs:

I usually avoid them. The language is terrible and they don’t address things.

User with medical conditions, age 84

Although we included a short phrase describing Blog Posts on the home page, many users glossed over it or did not recall reading it later in the session. Numerous users read the titles of the Blog Posts on the home page and expressed interest in the “articles.” Several users did not realize that the articles they were reading were Blog Posts. The original name for Web Resource Ratings, Web Product Rater, was misunderstood by a significant number of participants. The wording Browse Citizen Content was also unclear to some people who thought they could browse content that other citizen users had contributed.

Participants commented that optimal aging was an important issue for most of the population but access to the Portal would be limited to users who were computer literate, excluding many older users. In reaction to the Twitter feed at the bottom of the home page, most participants admitted that they did not use social networks such as Twitter or Facebook. Only 5 of 25 (20%) participants who talked about Twitter reported having an account, and only one was an active user. Participants found the Tweets interesting and reported they would read them but were not willing to join Twitter to do so.

**Challenges to usability.**

Reluctance to register: hesitation to register, caution about creating an account

Why would I have to register and log in if I wanted information? I would go to register and see what information was wanted or required. I am very careful about who I give my information to, and although this looks like a good, honorable website, I want to know why you require one to register to get information. [Caregiver, age 75]

I know for myself, as a person who doesn’t know the computer very well, that to just register is always a scary thing for me because I never know what it’s going to do to my computer. [Caregiver with medical conditions, age 59]

Registration process itself: obtaining a user account, following instructions

I want to be able to find what I am looking for. It is very annoying to go on even to register for something and then having to go from one screen to another, cross back. It is very frustrating. [User without medical conditions, age 59]

If registration is complicated and I have to make up a password, I would get frustrated and rethink whether I need the information. It has to be easy for me to register if I am going to continue. [User without medical conditions, age 50]

Searching: use of search function to navigate through the website, clarity of menus and instructions, learnability

I would not know what to search or where to search. I would be lost. It would be helpful if there was a dropdown list of overall subjects. Rather than think of what to search, I can choose something. [User without medical conditions, age 84]

I would browse first, because, for searching, I don’t know the context. [Caregiver, age 54]

Okay, enter search terms. I don’t know what I’m looking for. I assume it means type in something, for example, “exercise?” But that’s only an assumption, because that is what I'm interested in, so I would put that there. But when you use those terms, “Enter search terms” for a citizen who is not a medical practitioner or an academic, that might not be terms that they understand. [User with medical conditions, age 70]

Terminology: understanding of language and intentions on the website, use of jargon, names of features and functions

Blog has that connotation to me as being just anybody can go on and say something, whether it’s true or not. I am not interested in reading that kind of thing. [User with medical conditions, age 66]

So that is what I will call a unique use of the term. “Web Product” [original name of Web Resource Rating] to me, is a thing that you are selling. The term that is confusing is “product,” not Web, but product. [User with medical conditions, age 70]

“Connector”[original name of Blog Posts] is a weird term. [Caregiver with medical conditions, age 59]

Technology: user’s familiarity and use of technology, social media, user’s computer literacy

Seems like you need to know how to use the computer to use this portal. Some older people only use it for email. Therefore, simplicity is key. Some older users only started using the computer in the past few years. [Caregiver with medical conditions, age 59]

Of the people I know, nobody uses Twitter. [User without medical conditions, age 75]

I am not old old . . . but I am old enough in the sense that it has to be pretty obvious for me to continue; and if not, well the hell with it. [User with medical condition, age 61]

## Discussion

### Principal Findings

We evaluated an evidence-based health information website about optimal aging for health professionals and citizens. We assessed the overall Portal and 3 citizen-specific features (Evidence Summaries, Web Resource Ratings, and Blog Posts) by usability testing and individual interviews with citizen users. The evaluation findings revealed that the Portal met its goal of providing consumers with high quality, timely, practical information about aging. Therefore, the Portal can be recommended to patients, caregivers, and adults who are seeking reliable information on healthy aging and managing health conditions. We learned what citizens felt were the positive features of the website: credibility, applicability, browsing function, design, and accessibility. We also identified a number of usability challenges: reluctance to register, process of registering, searching, terminology, and technical features. These areas of concern were used to enhance the design and content of the citizen features of the Portal (Textbox 3).

**Usability challenges and modifications.**

Accessibility

- Kept the citizen content pages user friendly by minimizing the amount of text
- Moved additional written information to the About pages and Help section, accessible via links

Reluctance to register

- Moved text on “Why register?” so that it can be read before going to registration page

Registration process itself

- Removed the email validation step to activate and complete the registration process

Searching

- Included instructions and tips for searching, such as “What search terms do I use?” and “How are the search results ordered?”
- Improved query processing
- Added options for the display of search results (eg, order by highest rated, most accessed, most recent)
- Added spell check and word autocomplete features
- Ongoing work to improve and expand citizen friendly lexicon that addresses issues related to lay language synonyms, lay usage, and lay terms that cannot easily be mapped to medical vocabulary
- Increased volume of citizen friendly content

Terminology

- Revised the name of the Connector to Blog Posts
- Elicited feedback from participants about alternative wording for Web Product Rater and this led to the name being changed to Web Resource Ratings
- Provided additional labels and content description at the top of each citizen record
- Explanations of citizen content types were made more prominent on the Home page, About pages, and Help section
- Changed Browse Citizen Content to Browse Topics

Technology

- Made the video and clip buttons, including Play, visible at all times
- Made access to Tweets not reliant on having a Twitter account

### Limitations

The participants were a self-selected group of volunteers, which may limit generalizability. Participants had higher health literacy and computer literacy, which may have motivated them to enroll in the project. Most participants lived in socioeconomically well-established parts of the community. Our sample was also homogeneous with regard to ethnicity.

We were unable to recruit a significant number of younger seniors (aged 50-64 years), who are more likely to be interested in health promotion and disease prevention for themselves and health conditions for family members and friends, as informal caregivers. Older seniors (over 65 years) are more likely to search for health information on their own behalf compared to users aged 50 to 64 years [65]. A recent study found that baby boomers, born from 1946 to 1964, are more ready to use health information websites and employ smartphones for health care purposes compared to older adults [66]. This group is more likely to be in the workforce and have limited time to participate in usability research.

The sample was well educated, with 90% having at least a university degree, but survey research suggests that college graduates are the group most likely to search for online health-related information [65,67,68], so the study sample may well represent the target population.

The think-aloud method provides rich qualitative data from a small number of users, but the testing environment is likely to affect participant actions. We did our best to reduce social desirability bias. The facilitator introduced herself as an independent researcher who was not involved in the creation of the Portal and who welcomed negative feedback if it would lead to improvements and greater usage of the website.

Although the appropriate sample size to detect usability problems using the think-aloud method is debatable [69-72], we followed the recommendation of 10±2 participants for each testing method based on the meta-analysis by Hwang and Salvendy [51]. By increasing our numbers to 33 usability testers and 21 interview participants, we increased assurance that we found the difficulties that required attention [73]. However, using many of the same participants (43%) for both stages of testing may limit our findings.

By offering remote testing, we tried to include volunteers with mobility or distance issues that might prohibit their participation. While some researchers attest that remote synchronous testing is virtually equivalent to the conventional laboratory method [55,74,75], remote testing does have drawbacks. There was potential risk of bias in that testers using Skype were required to have a video-enabled computer and Internet connection with adequate bandwidth. Participants who were inexperienced with Skype were able to participate with coaching from the facilitator. Regardless, one user who was familiar with Skype experienced technical problems and was unable to share his screen. We were aware of potential difficulties using Skype such as dropped calls, inaudible audio, and frozen screens [61,76], but these technical problems were not experienced during the Skype sessions.

Overall, we found the use of Skype for usability testing and qualitative interviewing with older adults to be a feasible research medium and a practical alternative to face-to-face interviews. Increased bandwidth and the broad availability of Skype will help promote its use in health research. The video feature of Skype makes it preferable to telephone interviews. Participants can remain in their own personal space and use their own computer without losing interpersonal rapport and visual interaction [55,75]. Managing personal dynamics and developing trust between the facilitator and the participant is much more difficult over the telephone. There are also problems regarding the test setup and troubleshooting with telephone participants [77]. We found that some of our participants had difficulties with registration during telephone sessions. This could have been a result of poor usability, system problems, or miscommunication.

### Comparison With Previous Work

In this section, we describe generalizable implications of our results in the context of the research literature. Previous work has found that credibility and trustworthiness of health information are concerns for older online health seekers [78,79]. Users look for official branding when assessing the credibility of health information websites as well as information source, professional design, scientific basis, language, and ease of use [80,81]. Many of our participants told us that the McMaster University brand affirmed the Portal’s credibility. The lack of advertising may have also made an impact, because the presence of advertisements tends to decrease the credibility of Web pages [82,83].

Personal context is the main reason for accessing health information. Motivating factors include a new medical diagnosis (either personally or knowing someone who was recently diagnosed with a medical problem), a new prescription or treatment, coping with a chronic condition, and the decision to make lifestyle changes such as nutritional or exercise habits [84,85]. Seniors search the Internet for information on diseases or medical problems, symptoms, prescription drugs, and treatments [1,36,86]. Adults of all ages have gone online for information about nutrition, exercise, or weight control [87] and to prepare for doctor appointments [88,42,89]. These issues were also raised by participants using the Portal who reflected on topics that were relevant to their own lives.

Registering for an account on an information website was considered common to some of our participants, but many others (especially those who were older) had preliminary concerns about disclosing personal identifiers online. There is some research to suggest that older users find the registration process moderately complicated [90] and often forget to complete the final step in registering for an account on a patient portal [91]. Based on the usability findings, we eliminated the email validation step, which was the biggest barrier in the registration process.

Research suggests that tailored communication is more effective than nontailored messaging [85,92], and this effect is mediated by perceived message relevance [93]. To implement personalized features of the Portal such as tailoring of email alerts to topics of interest, registration is required. However, if the simplified registration process is perceived as a barrier to Portal use by older citizens, we may consider eliminating this requirement altogether.

The challenges that our participants faced with using the Portal’s search function reflect previous findings that most online users find articles through external search engines such as Google instead of using built-in search boxes [2,36]. Therefore, a website’s search method should be visually clear with specific instructions and few required steps [28]. As seen in previous research [94], our users were better at finding actual content (eg, locating a specific Evidence Summary or Web Resource Rating) than finding navigation tools (eg, locating the search box).

Citizens may struggle with their limited medical vocabulary when constructing a search query [28,95,96]. Query analysis research has shown that when people enter short queries into search engines for health information, it often leads to disappointment in the search results due to misrepresentation of the health problem [97] and an abundance of unwanted hits. Also, more than half of online seekers will not go beyond the first page of the search results [94,98]. This experience with using search engines may explain why our participants entered long queries (ie, more than three terms per query) into the Portal search box.

Searching for content was one of the usability tasks that did not meet our criteria for successful task completion. This is not surprising because our study’s participants were new to the Portal and introduced to it for testing rather than going to the Portal on their own as health information seekers. They might have chosen to explore the Portal using the browse function rather than using search to retrieve sought-after information, preferring an exploratory rather than a goal-directed approach to navigate the website [99,100]. Our usability protocol necessarily added artificiality to the experience of participants. In contrast, we anticipate that visitors to the Portal will be looking for specific content. As users become more familiar with the website’s content, we anticipate that the search function may become more useful. Other aspects of the Portal that deserve further exploration are usage of the filter and sort tools, links to related content, and email alerts.

Design aesthetics have been shown to positively impact the usability of websites [101]. Older users do not like screens that are busy and overloaded with information [47]. Since playing videos online may not be as familiar to users over the age of 60 years, clear labeling on function buttons (eg, Stop, Replay) is required. Once participants played the video on the Portal home page, their favorable responses made it clear that the video added to novice users’ understanding of the website.

When a Web page contains little white space, the information can appear dense, crowded, boring, and difficult to read [102]. This may have been the reason that many of our participants were unwilling to read through big blocks of text on some of the more informational Web pages (such as the About section). We found that the use of white space, subtitles, and chunking of information made the Evidence Summaries and Blog Posts very readable to our participants [45].

As pointed out by our participants, engaging with the Portal does come with requirements for its users: familiarity and experience using a personal computer and understanding of the Internet, its associated language, and technology [103]. Previous research has demonstrated that older adults are enthusiastic learners and need very little motivation to learn new technology [104,105].

In our study, 10 participants (27%) owned and used tablets. Of these, 2 users participated by phone using their tablets for usability testing. Computer tablet use has been increasing in older adults. A 2013 Pew survey found that 28% of adults aged 55 to 64 years and 18% of adults aged 65 years and older were tablet owners [106]. There is some evidence that seniors are very satisfied with the usability of tablets [107,108]. Older people may find tablets to be less complex, less technical, and less intimidating compared to conventional computers [109]. The Portal’s use of responsive Web design rather than fixed-width layout allows for the adaptation of the website’s content and appearance across devices of different sizes and capabilities (as opposed to needing a website specifically for each device) [110].

The use of social networking sites such as Facebook and Twitter is increasing in adults aged 50 years and older [111,112]. Unlike younger age groups whose primary use is for the purpose of socialization and fun, older adults and informal caregivers use social networking sites to gather and share information [3,113]. This is in keeping with our findings that participants did not use Twitter or Facebook due to their disinterest in online entertainment or social interaction but were very receptive to reading the Twitter feeds.

### Conclusions

We conducted usability testing to evaluate a continuously updated evidence-based health information website focused on healthy aging and management of health conditions. We also used individual interviews to evaluate 3 of its features (Evidence Summaries, Web Resource Ratings, and Blog Posts) with citizen end users. We employed usability evaluation to identify tasks that could be completed with ease (locating where to register, completing registration form, browsing the citizen friendly content, and locating specific resources) and tasks that required assistance (playing the video, validating the registration, searching, locating Blog Posts). The qualitative analysis of the interview transcripts revealed emerging themes that we organized into valued attributes and usability challenges. We learned what characteristics of the Portal are perceived as positive by citizens and should continue to be supported (credibility, applicability, browsing function, design, and accessibility). We also identified and addressed usability challenges (reluctance to register, registration process, searching, terminology, and technical features) to improve the overall Portal and its citizen friendly features.

This study reinforced the importance of including end users during the development of a dynamic, evidence-based health information website. Our findings can be applied by designers of health-related websites. Older adults are an important target audience as the population ages and increasingly adopts new technology. Online health information and Internet usability by older adults have become progressively relevant areas of research [114,115]. Our findings contribute to those bodies of work and can be applied by designers of health-related websites and producers of information resources for older users and informal caregivers. Further evaluation methods can be used to enhance the Portal and other health information websites for seniors: usability testing with professional groups and a larger sample of informal caregivers and younger age groups, usability testing on tablets and mobile devices, summative evaluation (usage analysis), assessment of user satisfaction, and impact of website engagement on health behaviors and outcomes. Future research should explore how health information websites can be integrated into systems of care, including technological linkages with medical systems.

## Appendix

Multimedia Appendix 1 McMaster Optimal Aging Portal: website technical design and development.
