# Supplementary material for: The McMaster Optimal Aging Portal: Usability Evaluation of a Unique Evidence-Based Health Information Website
Source: JMIR Hum Factors. 2016 May 11;3(1):e14. doi: 10.2196/humanfactors.4800 (PMC4880743; doi:10.2196/humanfactors.4800)
Supplement: Multimedia Appendix 1 [file humanfactors_v3i1e14_app1.pdf]

Multimedia Appendix 1

McMaster Optimal Aging Portal: Website Technical Design and Development

Software Design Framework

We used an iterative website design and development approach using “agile software development” methods (http://en.wikipedia.org/wiki/Agile_software_development). The Portal was built using a mobile first approach to ensure a stable responsive design that scales to any size phone, tablet or desktop. Our user experience focused on a ‘low click’ experience, helping the user to find the information they need as fast as possible.

Languages

A multilingual framework was used, allowing for a fully bilingual site in English and French, with potential for additional languages in the future.

Accessibility

We included the potential for CSS styles to accommodate higher contrast versions of the site for users with low vision. For older users who may have challenges using a mouse, we included more separation between hyperlinks, and button sizes were enlarged. Click zones or tap zones for touch interfaces were also generous in size to accommodate users with less dexterity. Quality assurance testing was done to ensure compatibility with a wide range of operating systems and browser versions.

Evidence-Based Content

Content for Professionals

The research evidence on the Portal intended for professionals comes from three best-in-class resources. McMaster Premium LiteratUre Service (McMasterPLUS™)[1,2] is an information service that provides the best research evidence for clinicians and includes articles that are continuously selected from more than 120 journals, through a critical appraisal process that identifies studies and systematic reviews that are scientifically strong. Articles that meet specific scientific criteria are then rated by frontline clinicians on two 7-point scales, one for clinical relevance and one for newsworthiness or novelty. The Health Evidence™ database,[3] developed for public health professionals, includes quality-rated systematic reviews about the effectiveness of public health interventions, such as immunization campaigns. The Health Systems Evidence database[4,5] was developed for managers and policymakers, and includes many types of research evidence about methods to address problems in the health system. The content from each of the three databases is filtered for relevance to healthy aging and health care for seniors (i.e., studies with older adults, research on health promotion or disease prevention or optimal management of health problems) before being included on the Portal.

Clinician Articles

The clinician articles, coming from McMasterPLUS™, contain the following:

- Title of the original article, authors, journal citation, and link to the abstract in PubMed
- Clinical peer ratings: ratings by three to four physicians from each pertinent discipline (33 possible clinical disciplines in total, e.g., internal medicine, cardiology, psychiatry) on relevance and on newsworthiness
- Scientific abstract
- Clinical comments provided by raters from each relevant discipline.

Public Health Professional Articles

The publichHealth articles, coming from Health Evidence™, include the following:

- Title of the original article
- Quality rating: Strong (8 to 10 / 10), moderate (5 to 7 / 10), or weak (1 to 4 / 10), assigned by two independent reviewers using the Health Evidence Quality Assessment Tool
- Full citation
- Links, if available: article full-text, abstract in PubMed, related podcast, Cochrane plain language summary, and related webinar
- Scientific abstract
- Keywords.

Policymaker Articles

The Policymaker articles, coming from Health Systems Evidence, are organized as follows for each document:

- Title of the original article
- Findings
    - Links to user-friendly summary, if available
    - Links to scientific abstract
    - Link to full-text report, if available for free
- Recency, quality and context of findings
    - Last year literature searched
    - Quality rating: assigned by the McMaster Health Forum using the AMSTAR rating
    - Countries in which studies (included in the synthesis) were conducted
- Additional details about the research
    - Type of document, e.g., systematic review of effects
    - Type of question, e.g., effectiveness, description
    - Focus, e.g., specific, general

- Priority area, if relevant
- Target, e.g., health system
- Health systems topic(s), e.g., financial arrangement, delivery arrangement, implementation strategy
- Theme, e.g. optima aging, health promotion/primary prevention
- Domain, by diseases, sectors, providers

- Publication details
  - Full citation
  - Digital object identifier (DOI).

Content for Citizens

The rigorous process used for inclusion of the professional resources in the Portal was also utilized to develop content for citizens, specifically Evidence Summaries and Blog Posts.

Evidence Summaries
The Evidence Summaries contain the following sections:

- Declarative title: key result (s) of the research study stated succinctly
- Descriptive title: subject of the study
- Research question: including population, interventions, setting, comparisons, and main outcomes if applicable
- Background: information as to the nature of the problem being addressed (including key technical terms and previous findings if available)
- How the review was done: including total number of studies and date ranges of studies (for systematic reviews); number of participants; funding sources (if relevant)
- What the researcher found: primary outcomes (main results); secondary outcomes; quality of evidence
- Definitions of key technical terms
- Links to the professional record.

These are examples of Evidence Summaries from each of the professional databases chosen by participants to evaluate:

- Flu vaccine reduces cardiovascular events (Clinical)
- Tests detect dementia in older people; cognitive stimulation or some drugs may slightly improve cognitive function (Clinical)
- Interventions to promote walking in groups increases physical activity in older adults (Public Health)
- Multiple lifestyle changes in people with established coronary heart disease reduce the risk for cardiovascular events (Public Health)

- Meaningful social roles may improve health and well-being for people in retirement (Policymaker)
- Unnecessary medication use in frail older adults can be reduced through team-based care, providing education to providers and reviewing prescribing practices (Policymaker).

Web Resource Ratings

For the Web Resource Ratings, the Portal provides the following:

- Link to the actual resource
- Overall star rating, from 1 (not recommended) to 5 (reliable information that we recommend discussing with your health care provider)
- Name of the website
- Summary of the Resource's content, including any information that you can act on related to optimal aging (actionable message)
- Resource type, e.g. article, podcast, fact sheet
- Star rating for each criterion: evidence-based (reliable information, based on scientific research), transparency (clear information about resource development), usability (easy to understand and use). These ratings are weighted to favor content that is based on scientific research evidence. Two staff members independently rate each resource. Any disagreements in ratings are discussed until consensus is reached.
- Links to related topics.

These are samples of the Web Resource Ratings evaluated during the interviews:

- Learn the facts about elder abuse and neglect
- Women and hair loss: Coping tips
- Breast cancer aware
- Men and bone density testing

Blog Posts

Blog Posts contain the following elements:

- Feature image about the topic being discussed
- Description of why the topic is important
- Summary of the research in the area or from a particular Evidence Summary
- Why the research findings are important
- Bottom line recommendations for citizens (in bullet points)
- References
- Author details
- Comments: users can comment on Blog Posts and view comments from other readers, if they have registered for the Portal and are logged in
- Links to related Blog Posts.

The Blog Posts chosen by our participants to review included:

- Sleep and aging: How many zzz's are optimal to stay healthy?
- Loneliness hurts. How to recognize loneliness as a health concern.
- How fast should I walk to cross the road safely? Fast facts about walking speed
- Want to become more physically active? Start by joining a walking group!

Mac_Aging News (Twitter feed)
The tweets are based on a 9 a.m. daily search of the previous day's media coverage. Each day, the Portal staff search for health and lifestyle news articles using Factiva in all Canadian newspapers and the Canadian Press, Canadian Press Broadcast and Postmedia newswires, and using Google News Alert. The search is based on the following key words: Aging, seniors, baby boomer, baby-boomer, Alzheimer, dementia, frail, frailty, ageing, elderly, homecare, home care, community care, and community-based care. One a list of potentially relevant articles is compiled, the Portal databases are searched for relevant evidence. We then tweet out the NEWS article paired with the related EVIDENCE tweets.

Other Content

There is also access to information about aging-related events such as expert-given public talks, live webcasts, as well as video recordings of these events.

Navigation and Content Retrieval

The Portal's overall organization, page design, font, icons and links have been designed and constructed to afford the user ease of navigation to its many features. The search engine powers the retrieval of Portal content, with features that both categorize and prioritize its search results. The results that best match the users' role are displayed most prominently in the central portion of the webpage while links to the Portal's other content are easily accessible in a sidebar menu. For example, for a "citizen search", the matching Evidence Summary citation and evidence rating are presented prominently for users to easily make selections on what to view in more detail. Other citizen content (Web Resource Ratings and Blog Posts), are available by links on the flanking portions of the page. Similarly, a search of the professional content retrieves and prioritizes content from the McMasterPLUS™, HealthEvidence™ and Health Systems Evidence databases in the main viewing frame, according to the user's chosen professional role. The related records from the other professional and citizen content available on the Portal are available on the side the page. For all search results, the number of items retrieved per citizen and professional content type are posted, and all search results in each category are ordered by evidence rating rank.

The Portal also offers the user a browsing option to find citizen content by way of selecting among 66 unique citizen topics organized into three categories which include: health conditions (e.g., back pain, cancer, stroke), healthy aging practice (e.g., exercise, nutrition) and healthcare delivery (e.g., community care). The browse feature allows the citizen to select a topic area of interest and retrieve all content indexed to the topic and listed in order of its quality of evidence. Citizen content tagged with a given category is connected to other similar content by following the topic link in the item's record, facilitating access to Evidence Summaries, Web Resource Ratings and Blog Posts on the topic. The citizen topic tags appear in all citizen content records and can be followed from a record also retrieved from a search.

References

1. Haynes RB, Holland J, Cotoi C, et al. McMaster PLUS: A Cluster Randomized Clinical Trial of an Intervention to Accelerate Clinical Use of Evidence-based Information from Digital Libraries. *J Am Med Informatics Assoc*. 2006;13(6):593-600. doi:10.1197/jamia.M2158.

2. Holland J, Haynes RB. McMaster Premium Literature Service (PLUS): an evidence-based medicine information service delivered on the Web. In: *AMIA Annual Symposium Proceedings*. ; 2005:340-344. PMID: 16779058.

3. Dobbins M, DeCorby K, Robeson P, Husson H, Tirilis D, Greco L. A knowledge management tool for public health: health-evidence.ca. *BMC Public Health*. 2010;10(1):496. doi:10.1186/1471-2458-10-496.

4. Lavis JN, Wilson MG, Moat KA, et al. Developing and refining the methods for a "one-stop shop" for research evidence about health systems. *Health Res Policy Syst*. 2015;13(1):10. doi:10.1186/1478-4505-13-10.

5. Moat K a., Lavis JN. 10 Best Resources for ... Evidence-Informed Health Policy Making. *Health Policy Plan*. 2013;28(2):215-218. doi:10.1093/heapol/czs050.
